# Antibiotic Recommendations for Treatment of Canine Stromal Corneal Ulcers

**DOI:** 10.3390/vetsci10020066

**Published:** 2023-01-17

**Authors:** Milan Joksimovic, Bradley A. Ford, Tatjana Lazic, Ivan Soldatovic, Sergey Luzetsky, Sinisa Grozdanic

**Affiliations:** 1 Animal Eye Consultants of Iowa, 698 Boyson Road, Hiawatha, IA 52233, USA; 2 Department of Pathology, Roy J. and Lucille A. Carver College of Medicine, University of Iowa, Iowa City, IA 52242, USA; 3 Oculus–Ophthalmology Specialty Hospital, 11000 Belgrade, Serbia; 4 Department of Biostatistics, College of Medicine, University of Belgrade, 11000 Belgrade, Serbia

**Keywords:** corneal, stromal, ulcer, bacterial, canines, antibiotic

## Abstract

**Simple Summary:**

Infection of the cornea is among the most frequent causes for the loss of vision in dogs. The purpose of this study was to determine which particular antibiotics can be used immediately at the time of infection to eliminate bacteria from the infected region and prevent the loss of the eye. This study showed that combinations of antibiotics (amikacin and neopolybac or ofloxacin and neopolybac) are potentially the best first choice of treatment to eliminate the majority of commonly isolated bacteria from corneal infections in dogs.

**Abstract:**

The aim of the study was to identify the aerobic bacterial isolates and determine corresponding antibiotic susceptibility profiles in vitro in canine clinical specimens with stromal corneal ulcers, with the goal of providing recommendations for first-line treatment with antibiotics. A total of 198 canine corneal stromal ulcer samples were studied between 2018 and 2021. A corneal swab was collected and cultured under aerobic conditions. Bacterial organisms were identified at the species level by MALDI-TOF mass spectrometry. Antibiotic susceptibility testing for commonly used topical and systemic antibiotics was performed by disk diffusion. Bacterial growth was obtained from 80% of samples. A variety of bacterial species were identified wherein the most common specimens were represented by *Staphylococcus pseudintermedius* (22%), *Staphylococcus epidermidis* (12%), *Staphylococcus capitis* (11%), and *Pseudomonas aeruginosa* (10%). Based on the overall antibiotic susceptibility data, neopolybac alone (96%) or a combination of neopolybac with either ofloxacin or amikacin (each 99%) showed the best coverage for commonly isolated bacterial organisms from canine corneal stromal ulcers. Results of this study support the use of the combined antibiotics as the first-line response for the treatment of canine corneal stromal ulcers. A statically significant increase in acquired bacterial resistance was detected during the longitudinal data observation.

## 1. Introduction

The ocular surface is constantly exposed to a variety of environmental stimuli and contains different mechanisms which function as a first level of eye defense against possible pathogens. Bacteria often invade the damaged corneal surface, which in turn may lead to the acceleration of corneal tissue loss, resulting in structural integrity defects and potential loss of the eye [1,2]. The consequences could be vision-threatening and devastating for eye globe integrity if the corneal infection process is not immediately and aggressively treated or the causative bacterial organism is resistant to empiric antibiotic treatment [3]. The first step in treating corneal bacterial infections is empiric therapy based on epidemiological data and use of suggested antimicrobials [4]. While large epidemiological and corneal pathogen surveillance studies have been reported in humans, similar datasets are relatively sparsely reported in veterinary medicine, so the initial selection of the antibiotic treatment is frequently chosen based on personal preference and in-hospital ophthalmic drug availability [4,5,6,7,8,9,10].

A number of studies have evaluated a microbial community in canine corneal ulcers [5,6,7,8,9,10,11,12,13,14,15]. The most frequent bacterial groups identified are Gram-positive staphylococci and streptococci in addition to Gram-negative *Pseudomonas aeruginosa* [3,5,7,8,9,11,12,13,14,16,17].

The primary purpose of this manuscript was to perform in depth analysis of antimicrobial activity for commonly identified bacteria from canine corneal stromal ulcers and provide general guidelines for the immediate initiation of empiric antibiotic therapy, which may have the highest chance of being effective while waiting for the results of laboratory microbial identification and antibiotic susceptibility. Furthermore, we intended to evaluate trends in the antibiotic resistance development over a four-year period with a goal of providing predictive data for future topical antibiotic use for canine corneal stromal ulcers.

## 2. Materials and Methods

Canine corneal ulcer samples were harvested using a flocked swab kit and placed in the provided transport media (BD ESwab^TM^ Collection Kit, COPAN ITALIA SpA, Brescia, Italy). All samples were collected 30 s after applying topical anesthetic on the ocular surface by gently rolling over the corneal surface for 10 s (Propracaine 0.05%, Akorn Pharmaceuticals, Lake Forest, IL, USA). The samples were then kept on ice packs until submitted to the laboratory or refrigerated at 4 °C and subsequently cultured after 1 to 5 days of collection.

Bacterial swabs were collected in the period from December 2018 to April 2021 from canine patients with corneal stromal ulcers presented to Animal Eye Consultants of Iowa in the state of Iowa, USA. All patients had a complete eye examination. The inclusion criteria for corneal stromal ulcers were presence of the corneal defect affecting at least 10% of the corneal stromal thickness with the clinical signs of cellular neutrophilic infiltrates with or without evidence of corneal melting. Half of each ESwab tube solution with a collected sample (approximately 0.5 mL) was cultured onto MacConkey agar (Hardy Diagnostics, Santa Maria, CA, USA), while the other half of the sample was cultured onto Chocolate agar (Hardy Diagnostics Hardy Diagnostics, Santa Maria, CA, USA). The plates were incubated at 37 °C in 5% CO_2_ and examined at 24, 48, and 96 h after plating for bacterial colonies.

Antibiotic susceptibility testing was performed by Kirby–Bauer disc diffusion method in all isolates following Clinical and Laboratory Standards Institute (CLSI) guidelines (https://clsi.org/media/3481/m100ed30_sample.pdf, accessed on 10 January 2023 and https://clsi.org/media/2321/vet08ed4_sample.pdf, accessed on 10 January 2023). An ophthalmology antibiotic panel was developed based on the most frequently used and commercially available topical ophthalmic antibiotics in the midwestern US. Additionally, amoxicillin/clavulanic acid was added to the panel as this antimicrobial is frequently used as a systemic antibiotic after different ophthalmic surgeries. The topical antibiotic set included amikacin (30 µg), bacitracin (10 U), cefazolin (30 µg), cefoxitin (30 µg), gentamicin (10 µg), neomycin (30 µg), ofloxacin (5 µg), oxacillin (1 µg; used instead of cefoxitin in the case of *Staphylococcus pseudintermedius* and *Staphylococcus schleiferi* per CLSI guidelines; https://clsi.org/media/2321/vet08ed4_sample.pdf, accessed on 10 January 2023), polymyxin B (300 U), tetracycline (30 µg), and tobramycin (10 µg). The systemic antibiotic set included amoxicillin/clavulanic acid (20/10 µg), cephalexin (30 µg), ciprofloxacin (5 µg), clindamycin (2 µg), doxycycline (30 µg), enrofloxacin (5 µg), marbofloxacin (5 µg), penicillin G (10 U), and sulfamethoxazole/trimethoprim (1.25/23.75 µg). *Escherichia coli* ATCC 25922 and *Staphylococcus aureus* ATCC 25923 were the quality control organisms. All antibiotic discs and control cultures were provided by Hardy Diagnostics (Santa Maria, CA, USA) and Microbiologics INC (St Cloud, MN, USA), respectively.

Bacteria were identified to the species level by matrix-assisted laser desorption/ionization-time of flight (MALDI-TOF) analysis following manufacturer’s instructions (Bruker, Madison, WI, USA). Briefly, a single colony no older than 5 days was taken from the culture plate with a toothpick. On the target plate, a thin bacterial layer was smeared onto a single spot and then the same specimen was placed onto the next spot to achieve a thinner bacterial layer. Each sample was covered with 1 µL of 100% formic acid and air dried and then 1 µL of HCCA matrix was added to each spot as instructed by manufacturer. Bacterial Test Standard, *Escherichia coli* ATCC 25922, *Enterococcus faecalis* ATCC 29212, and *Candida albicans* ATCC 10231 were used as quality controls (Microbiologics, INC; St Cloud, MN, USA). MALDI-TOF analysis was performed in a CLIA-certified diagnostic laboratory (Clinical Microbiology Laboratory, University of Iowa Hospitals & Clinics, Iowa City, IA, USA) using the Bruker BioTyper RUO Database which included continuously updated versions of the Compass reference library (in which veterinary isolates are well represented) as well as the optional mycobacterial and fungal libraries.

WHONET database software (World Health Organization) was used with 2022 CLSI breakpoints for dogs. If breakpoints were not available for dogs, other CLSI animal breakpoints were used followed by human CLSI breakpoints if no other animal breakpoints were available. These breakpoints were based on CLSI M100 Performance Standards for Antimicrobial Susceptibility Testing (https://clsi.org/media/3481/m100ed30_sample.pdf, accessed on 10 January 2023), CLSI VET08 Performance Standards for Antimicrobial Disk and Dilution Susceptibility Tests for Bacteria Isolated From Animals (https://clsi.org/media/2321/vet08ed4_sample.pdf, accessed on 10 January 2023), and Hardy Diagnostics Disk Diffusion Zone Diameter Chart (https://www.keyscientific.com/files/Other%20Manufacturers/Hardy%20Diagnostics/AST%20Discs/Hardy%20AST%20Disc%20Insert.pdf, accessed on 10 January 2023). WHONET database software was also used to manage and analyze microbiology laboratory data and antibiotic susceptibility test results. Hardy Diagnostics Disk Diffusion Zone Diameter Chart https://www.keyscientific.com/files/Other%20Manufacturers/Hardy%20Diagnostics/AST%20Discs/Hardy%20AST%20Disc%20Insert.pdf, accessed on 10 January 2023) was also used for polymyxin B breakpoints in *Pseudomonas aeruginosa* isolates (Resistant ≤ 11; Susceptible ≤ 12).

The Alere^TM^ PBP2A SA Culture Colony Test was performed to detect penicillin-binding protein 2A (PBP2A) in staphylococcal isolates according to manufacturer’s instructions (Alere Scarborough, Inc., 10 Southgate Road, Scarborough, ME 04074, USA). In the case of *Staphylococcus pseudintermedius* and *Staphylococcus schleiferi* the methicillin resistance was confirmed with oxacillin disks per CLSI VET08 guidelines (https://clsi.org/media/2321/vet08ed4_sample.pdf, accessed on 10 January 2023).

All bacterial isolates were separated into susceptible and resistant categories according to the interpretive criteria above. Susceptible and intermediate levels of response were assigned to the susceptible class for the purposes of antibiogram creation [5,6,10].

Each antibiotic was classified into the antibiotic categories of aminoglycosides (amikacin, gentamicin, neomycin, and tobramycin), polypeptides/polymyxins (bacitracin, polymyxin B), anti-staphylococcal β-lactams (cephamycins, oxacillin, cefoxitin), tetracyclines (tetracycline), non-extended spectrum cephalosporins (1st and 2nd generation cephalosporins, cefazolin), penicillin and β-lactamase inhibitors (amoxicillin/clavulanic acid), and fluoroquinolones (ofloxacin). Each clinical isolate was classified by group based upon its susceptibility data according to resistance pattern as not multidrug-resistant (Not MDR), multidrug-resistant (MDR), or possible extensively multidrug-resistant (possible XDR). The MDR group was defined as resistance to at least one antibiotic in three or more antibiotic categories. XDR was defined as resistant to at least one antibiotic in all but two or fewer antibiotic categories (i.e., bacterial isolates remain susceptible to only one or two categories) as previously proposed [18,19]. Additionally, intrinsic resistance of an isolate to a particular antibiotic was excluded from this analysis as previously suggested [20].

Statistical analyses were performed using a paired *t*-test and contingency table analyses (chi-square and Fisher’s exact tests) for the indicated observed parameters with commercial software as described in the manuscript (Prism, version 5.0; GraphPad, San Diego, CA, USA).

## 3. Results

### 3.1. Bacterial Growth from Patient Samples

A total of 187 dogs (198 eyes) with corneal stromal ulcers were subjected to sample collection and a total of 198 samples were plated; 159/198 (80.3%) of plated samples demonstrated bacterial growth, while 39/198 (19.7%) yielded no growth. A total of 167 isolates were collected. Regarding prior antibiotic exposure, 134/198 (67.7%) samples were collected from patients having previous antibiotic treatment, while 64/198 (32.3%) samples were collected from patients with no previous antibiotic treatment; 101 of 134 (75.4%) plated samples resulted in the growth of isolates, while 33/134 (24.6%) yielded no growth in the group of patients with previous antibiotic treatment. Regarding patients with no previous antibiotic treatment, 54/64 (84.4%) plated samples returned growth of isolates while 10/64 (15.6%) resulted in no isolate growth.

### 3.2. Distribution of Bacterial Species in Patients Diagnosed with Corneal Stromal Ulcers

The most common bacterial species identified in the corneal stromal samples was *Staphylococcus pseudintermedius* present in 22% of samples. *Staphylococcus epidermidis*, *Staphylococcus capitis*, and *Pseudomonas aeruginosa* were present in 12%, 11%, and 10% of samples, respectively. *Enteric Gram-negative rods*, *coagulase negative staphylococci*, and *Streptococcus canis* were present in 7%, 5%, and 5% of samples, respectively (Table 1).

### 3.3. Antibiotic Susceptibility Pattern of Bacteria Isolated from Corneal Stromal Ulcers

**Figure 1:**
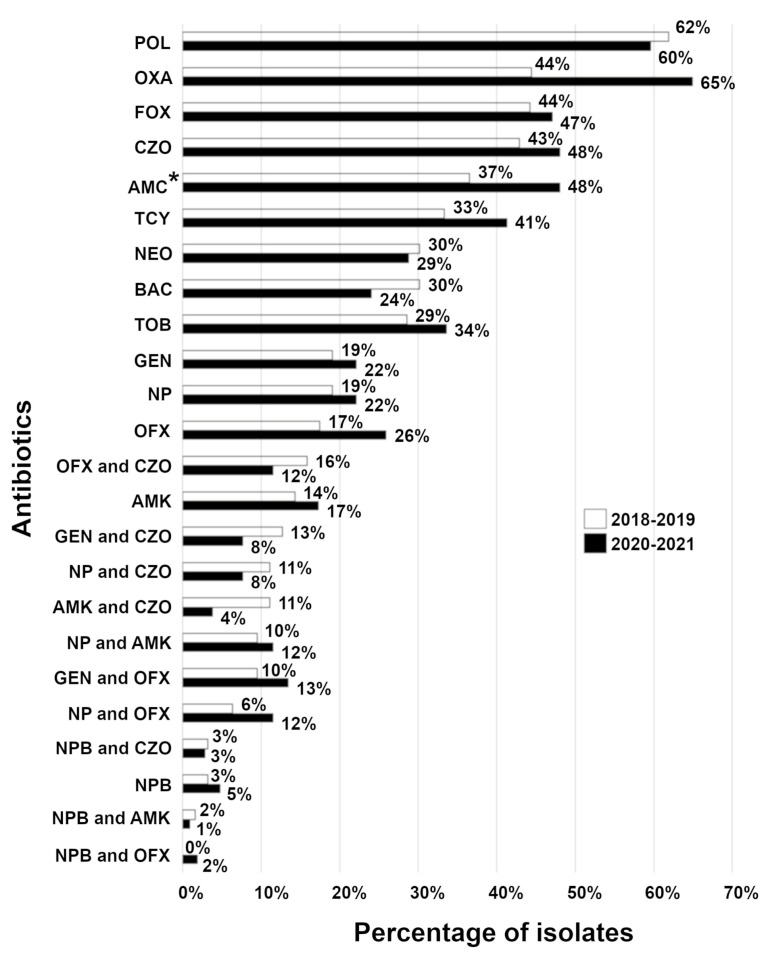
Percentage of total resistant isolates to a single or combination antibiotics from 2018–2019 and 2020–2021. Percentage (%) of overall resistant bacterial species isolated from patients with corneal stromal ulcers (combined intrinsic and acquired resistance). For the period 2018–2019, the number of isolates is 63 for each antibiotic/antibiotic combination except for oxacillin, which numbered 9. For the period 2020–2021, the number of isolates is 104 for each antibiotic/antibiotic combination except for oxacillin, which numbered 20. * = although amoxicillin/clavulanic acid is not used topically, data for this antibiotic are presented here since it is often used postoperatively as a systemic antibiotic. Abbreviations: AMC, amoxicillin/clavulanic acid; AMK, amikacin; BAC, bacitracin; CZO, cefazolin; FOX, cefoxitin; GEN, gentamicin; NEO, neomycin; NP, neomycin and polymyxin B; NPB, neomycin, polymyxin B, and bacitracin; OFX, ofloxacin; OXA, oxacillin; POL, polymyxin B; TCY, tetracycline; TOB, tobramycin.

To gain insight into which antibiotics to use in treating current canine corneal stromal ulcers, we analyzed resistance profiles of isolates relative to a single or combination antibiotic between two time points, 2018–2019 and 2020–2021. Based on overall antibiotic susceptibility data, neopolybac alone (96%) or a combination of neopolybac with either ofloxacin or amikacin (each 99%) showed the best antibiotic coverage for commonly isolated bacterial organisms from canine corneal stromal ulcers (Figure 1). No statistically significant difference (*p* = 0.1637, paired *t*-test) was observed in bacterial resistance to a single or combination antibiotic between these two time points (Figure 1). For all samples tested with topical antibiotics, bacterial species were most frequently resistant to polymyxin B, oxacillin, cefoxitin, and cefazolin. The least resistance was detected against amikacin, gentamicin, and ofloxacin (Table 2). When acquired resistance was analyzed, a similar trend of resistance was observed (Table 2).

For all samples tested with systemic antibiotics, bacterial species were predominantly resistant to penicillin G (74%), cephalexin (65%), clindamycin (59%), amoxicillin/clavulanic acid (44%), ciprofloxacin (41%), sulfamethoxazole/trimethoprim (41%), and doxycycline (34%), while the least resistance was seen against enrofloxacin (21%), and marbofloxacin (15%).

### 3.4. Increase in Acquired Resistance in Isolates from Corneal Stromal Ulcers

**Figure 2:**
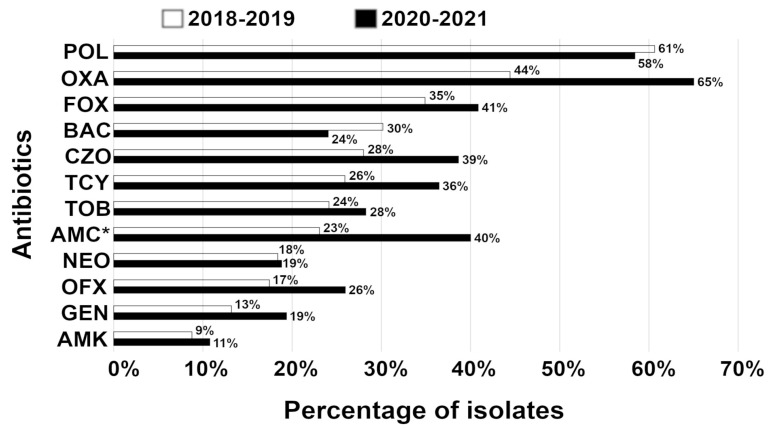
Increase in acquired resistance in isolates from corneal stromal ulcers between 2018–2019 and 2020–2021. Percentage (%) of resistant bacterial species from patient samples excluding intrinsic resistance of isolates. The number of isolates ranges from 9 to 63 for the period 2018–2019 and from 20 to 104 for the period 2020–2021. * = although amoxicillin/clavulanic acid is not used topically, data for this antibiotic are presented here since it is often used postoperatively as a systemic antibiotic. Abbreviations: AMC, amoxicillin/clavulanic acid; AMK, amikacin; BAC, bacitracin; CZO, cefazolin; FOX, cefoxitin; GEN, gentamicin; NEO, neomycin; OFX, ofloxacin; OXA, oxacillin; POL, polymyxin B; TCY, tetracycline; TOB, tobramycin.

To gain insight into the temporal dynamics of acquired resistance, we analyzed isolates between two time points, 2018–2019 and 2020–2021. In the period 2018–2019, the highest percentage of isolates was resistant to polymyxin B, oxacillin, and cefoxitin with a similar trend in the period 2020–2021 (Figure 2). The lowest percentage of isolates was resistant to amikacin, gentamicin, ofloxacin, and neomycin in the period 2018–2019 with a comparable tendency in the period 2020–2021. Strikingly, a temporal increase in acquired resistance was statistically significant (*p* = 0.0025, paired *t*-test) from 2018–2019 to 2020–2021 (Figure 2).

### 3.5. Distribution of Bacterial Species in Patients Diagnosed with Corneal Stromal Ulcers Relative to Previous Patient’s Antibiotic Treatment

The most common bacterial species identified in the corneal stromal samples relative to the patient’s previous antibiotic treatments were Staphylococcus pseudintermedius, Staphylococcus epidermidis, Staphylococcus capitis, and Pseudomonas aeruginosa (Table 3). When compared to the overall data in Table 1, most of isolates were present in comparable percentages. Accordingly, no statistically significant difference (*p* = 0.3367, paired *t*-test) was observed in the distribution of bacterial species relative to the patient’s previous antibiotic treatments.

### 3.6. Susceptibility Profile of Isolates from Corneal Stromal Ulcers Relative to Patient’s Previous Antibiotic Treatments

**Figure 3:**
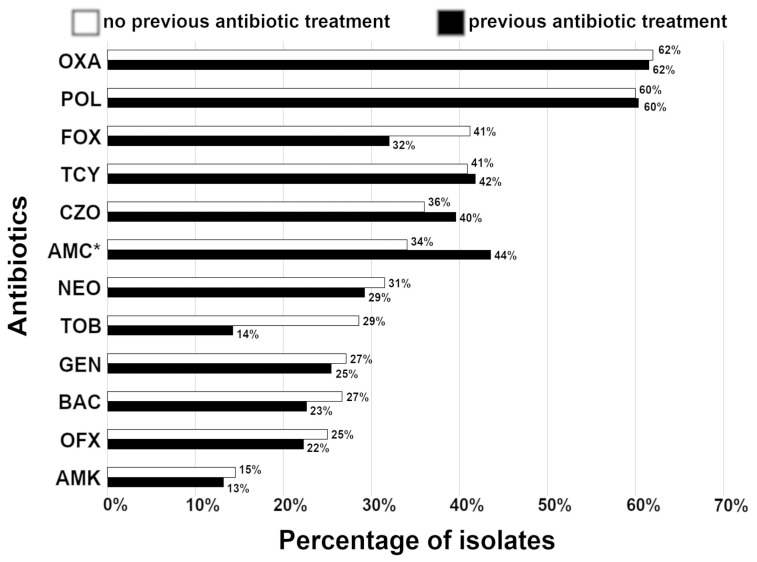
Acquired resistance in isolates from corneal stromal ulcers regarding the patient’s previous antibiotic treatments. Percentage (%) of resistant bacterial species against antibiotics excluding intrinsic resistance of isolates. The number of isolates range from 16 to 60 for the patients without previous antibiotic treatments and from 13 to 106 for the patients with previous antibiotic treatments. * = although amoxicillin/clavulanic acid is not used topically, data for this antibiotic are presented here since it is often used postoperatively as a systemic antibiotic. Abbreviations: AMC, amoxicillin/clavulanic acid; AMK, amikacin; BAC, bacitracin; CZO, cefazolin; FOX, cefoxitin; GEN, gentamicin; NEO, neomycin; OFX, ofloxacin; OXA, oxacillin; POL, polymyxin B; TCY, tetracycline; TOB, tobramycin.

To examine a trend of acquired resistance relative to previous the patient’s antibiotic treatments, we analyzed isolates within an approximately three-year period from 2018 to 2021. In the group with no previous exposure to antibiotics, the highest percentage of isolates was resistant to oxacillin, polymyxin B, cefoxitin, and tetracycline with a similar trend in the group previously treated with antibiotics (Figure 3). The lowest percentage of isolates was resistant to amikacin and ofloxacin in both groups. Furthermore, no statistically significant changes (*p* = 0.0977, paired *t*-test) in acquired resistance were observed relative to the patient’s previous antibiotic treatments within the examined three-year period (Figure 3). Furthermore, we analyzed total isolate resistance against the most commonly prescribed antibiotics by local non-specialty veterinary practices, ofloxacin and tobramycin, relative to the patient’s previous antibiotic treatments. In either case, no statistically significant changes in resistance were observed relative to previous patient’s antibiotic treatments (ofloxacin; odds ratio = 1.744; CI= 0.3717–8.647, *p* = 0.7163; tobramycin (odds ratio = 0.2857; CI = 0.05942–1.240, *p* = 0.1919; contingency table analyses (chi-square and Fisher’s exact tests)).

### 3.7. Resistance Profile Based on the Percentage of Isolates Resistant to Multiple Antibiotics in Canine Corneal Stromal Ulcers

**Figure 4:**
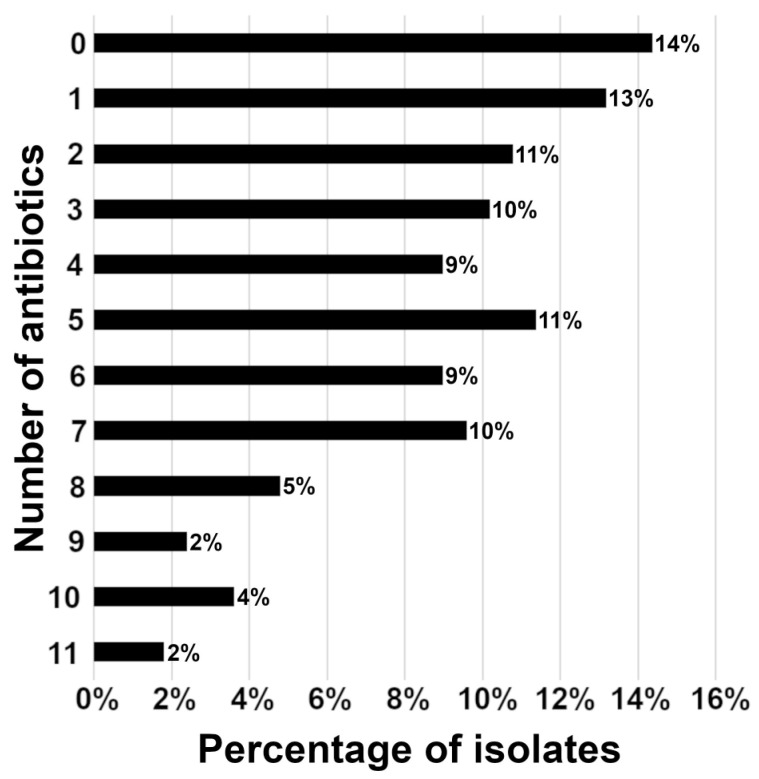
Distribution of resistant isolates against multiple antibiotics. Percentage of the isolates that are resistant to the corresponding number of antibiotics. Bacterial samples were isolated from canine corneal stromal ulcers (number of isolates = 167).

**Figure 5:**
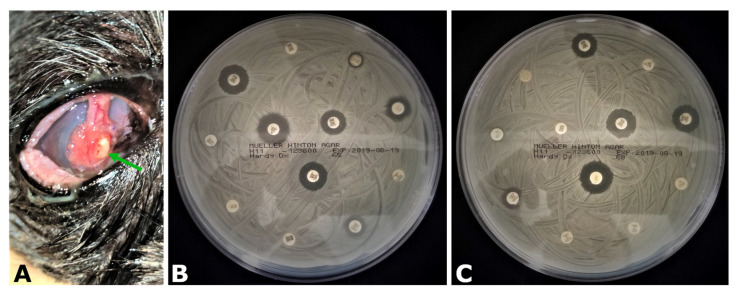
Aggressive corneal infection by *Staphylococcus pseudintermedius*. (**A**) Patient cornea was severely affected with a focal zone of conjunctival graft destruction at 4 o’clock position in the paracentral graft region (green arrow). Patient was treated with topical ofloxacin and cefazolin and systemic amoxicillin/clavulanic acid immediately after surgery. (**B**,**C**) Antibiotic susceptibility testing performed for topical (**A**) and systemic antibiotic sets (**B**). The isolate was resistant to the multiple topical (amikacin, cefazolin, cefoxitin, gentamicin, neomycin, ofloxacin, polymyxin B, tetracycline, and tobramycin) and systemic antibiotics (amoxicillin/clavulanic acid, cephalexin, ciprofloxacin, clindamycin, doxycycline, enrofloxacin, marbofloxacin, penicillin G, and sulfamethoxazole/trimethoprim). The isolate was also penicillin-binding protein 2A–positive and susceptible only to topical bacitracin.

The highest percentage of bacteria was not resistant to any tested antibiotic (24/167; 14%) followed by bacteria resistant to one antibiotic (22/167; 13%), while the vast majority of isolates (73%) were resistant to two or more tested antibiotics (Figure 4). Furthermore, no statistically significant difference (*p* = 0.1855, paired *t*-test) was observed in the percentage of isolates resistant to multiple antibiotics relative to the patient’s previous antibiotic treatments. Some highly aggressive isolates showed antibiotic resistance to nine or more tested antibiotics (Figure 4), such as a particular case of *Staphylococcus pseudintermedius* (Figure 5).

### 3.8. Distribution of Multidrug-Resistant (MDR) Bacteria in Clinical Corneal Ulcers

Overall, in the corneal stromal ulcer samples, 62% (103/167) were not MDR isolates, 20% (33/167) were MDR isolates, while 18% (31/167) were possible XDR isolates from 2018–2021. Regarding the patient’s previous antibiotic treatments, no statistically significant difference (*p* = 0.4325, paired *t*-test) was observed in the distribution of multidrug-resistant bacteria. In the period 2018–2019, 67% (42/63) were not MDR isolates and 19% (12/63) were MDR isolates, while 14% (9/63) were possible XDR isolates. In the period 2020–2021, 59% (61/104) were not MDR isolates and 20% (21/104) were MDR isolates, while 21% (22/104) were possible XDR isolates. Moreover, no statistically significant difference (*p* = 0.2777, paired *t*-test) was observed in multidrug resistance between these two time points.

### 3.9. Methicillin-Resistant Staphylococcus spp.

To assess methicillin resistance in *Staphylococcus* species, the isolates were tested with cefoxitin disks [21]. A few species were also tested with the penicillin-binding protein 2A (PBP2A) antibody test. Overall, in the 2018–2021 period, 49% (40/81) of isolates were methicillin-resistant. Relative to the patient’s previous antibiotic treatments, similar percentages of resistance were detected. In the group with no previous antibiotic treatment, 54% (15/28) of isolates were methicillin resistant. In the group with previous antibiotic treatment 47% (25/53) of isolates were methicillin resistant. Over time, a substantial increase in the number of methicillin-resistant *Staphylococcus* species were detected from 2018–2019 (39%; 11/28) to 2020–2021 (55%; 29/53).

## 4. Discussion

In this study, the overall bacterial growth from patients with corneal stromal ulcers (80.3%) was generally higher than previously reported rates (57–71%) [5,7,8,10,13,22,23]. The higher positive culture rate in our study may reflect the use of improved elution swabs (BD ESwab^TM^ Collection Kit) and possibly be due to increased yield of plating half of the volume of each collection swab onto a culture plate rather than a subset of the total volume adsorbed onto the swab.

The most common bacterial isolates from the stromal corneal ulcers in our study were *Staphylococcus* spp. accounting for 50% of all bacterial species, consistent with or slightly above levels reported in previous studies that were performed in various geographical locations (Table 4) [5,6,8,9,11,13,15,22,24,25,26]. In contrast, studies from Australia and UK reported *Pseudomonas aeruginosa* and *Streptococcus* spp. as the most commonly isolated bacteria from canine ulcers (Table 4) [7,10]. This discrepancy could be potentially explained by regional differences of bacterial species in various geographical locations due to local climate factors [4,9], wherein weather conditions in the midwestern US are not as warm and humid as in the southeastern US, Brazil, or Australia (Table 4).

Based on the overall antibiotic susceptibility data, neopolybac alone (96%) or a combination of neopolybac with either ofloxacin or amikacin showed the best coverage for commonly isolated bacterial organisms from canine corneal stromal ulcers in line with a previous report [15]. Considering that canine stromal corneal ulcers may be extremely aggressive, an immediate and aggressive initiation of antibiotic therapy with commercially available ophthalmic antibiotics (neopolybac and ofloxacin) may be the prudent strategy while waiting for the results of the microbial identification and susceptibility from the affected patient.

Data from this study described a trend of increased resistance to polymyxin B and ofloxacin when compared to previous studies [5,10,12]. However, a comparison of data from this study to the recent report [15], both performed at the same general geographical location (midwestern US), revealed substantial differences in topical susceptibility profiles for polymyxin B, bacitracin, and cefazolin (Table 5). This discrepancy can be partly explained by different methods used in these two studies (Kirby–Bauer disc diffusion method vs. minimum inhibitory concentration (MIC) susceptibility testing). Since the reliability of bacterial resistance to polymyxin B assessed by the Kirby–Bauer disc diffusion method is still questionable [27,28], reported polymyxin B data should be carefully scrutinized when making the clinical judgement on the choice of antibiotic. The same logic can be applied for bacitracin as this antibiotic, together with polymyxin B, belongs to the same antibiotic group of polypeptides. The trend of increased resistance to ofloxacin may reflect the acquisition of mutations through mobile genetic elements as reported in the case of *Pseudomonas aeruginosa* [29].

In our study, a patient’s previous antibiotic treatments do not affect isolate resistance to tobramycin in general. However, resistance to tobramycin appears to be increased between two time points, in line with a previous report performed at the same general geographical location [15] (Table 5; midwestern US). In contrast, the other study performed in the southeastern US demonstrated a substantially higher increase in and the percentage of resistant isolates to tobramycin [6]. As previously discussed, this inconsistency could be due to local climate factors in conjunction with the regional variation of bacterial species [4,9].

In the cases where severe corneal neovascularization is present or conjunctival pedicle graft surgery was performed so iatrogenic blood supply can be provided to the corneal ulcer region, treatments with topical medications can be complemented by systemic antibiotics. Based on data reported in this study, a systemic fluoroquinolone antibiotic (marbofloxacin, enrofloxacin) should be the first choice for treatment of corneal stromal ulcers, which is in line with a recently published report from the US Midwest on corneal ulcers [15]. In comparison to the earlier report from the southeastern US [6], in this study there was a tendency of increased resistance to enrofloxacin and ciprofloxacin, although this difference may be caused by regional differences in a distribution of bacterial species at different geographical locations and local climate factors [4,9].

In this study, we report a statistically significant increase in acquired resistance in isolates from 2018–2019 to 2020–2021. Our data not only point to this alarming trend but also indicate a presence of detectable deteriorating changes in antimicrobial susceptibility within a relatively short three-year period. However, the presence or absence of previous antibiotic treatments does not appear to influence an overall status of acquired bacterial resistance.

In our study, we detected 8% of isolates which showed antibiotic resistance to nine or more tested antibiotics. This pattern is of particular concern and qualifies corresponding isolates as potentially very aggressive pathogens causing corneal pathology poorly responsive to medical and surgical treatments.

The surge in antibiotic resistance is an alarming concern not only in global health care but also in animal ophthalmology [30]. In this study, over a third of isolates from clinical corneal stromal ulcers belong to the MDR group. However, none of *Pseudomonas aeruginosa* specimens belong to the MDR class, consistent with a previous report [15]. This study did not detect a statistically significant MDR increase between two time points 2018–2019 and 2020–2021 in contrast to the previous report, suggesting an MDR increase over time period of 2016–2020 [15].

Methicillin resistance of *Staphylococcus* spp. isolates is a serious concern in human and veterinary medicine due to the cross-species infectious behavior of these bacteria [31,32]. In this study, a half of *Staphylococcus* spp. isolates were methicillin resistant, which is in line with a previous report [33] with some isolates showing extremely aggressive clinical behavior (Figure 5).

In conclusion, the current study reports four important findings directly relevant to antibiotic treatments of canine corneal stromal ulcers: (1) clinical corneal stromal isolates showed increased acquired resistance within a three-year period; (2) many isolates were resistant to a large number of antibiotics; (3) over a third of analyzed specimens belong to the multidrug resistance group; and (4) some clinical isolates showed resistance to a combination of up to four antibiotics. Similar data have been recently reported in the ARMOR study from human corneal isolates; however, analysis of the resistance trend did not show gradual progression over a period of 10 years [4].

Analogous to earlier studies of antibiotic susceptibility in animals, key limitations of this study are the fact that CLSI interpretive criteria and breakpoints for particular bacterial species and antibiotic combinations are based on systemic minimum inhibitory concentration of antibiotics, since specific standards for corneal infections were never developed in human or veterinary medicine. Consequently, complete reliability of the Kirby–Bauer disc diffusion system as a method to assess corneal infections can be fully evaluated when these standards are developed. Until specific ophthalmology antibiotic standards become available, this study may provide a general guideline when initially choosing empirical therapies for treating canine corneal stromal ulcers while waiting for the patient-specific antibiotic susceptibility profile.

## 5. Conclusions

The results of this study support the use of the combined antibiotics as the first-line response for the treatment of canine corneal stromal ulcers. Neopolybac alone or a combination of neopolybac with either ofloxacin or amikacin is recommended as the initial antibiotic treatment while waiting for the patient-specific antibiotic susceptibility profile.

## Figures and Tables

**Table 1:** Bacterial species distribution from corneal stromal ulcer samples. Distribution of incidence of the specific microorganism detection in canine patients. Data are presented as a percentage of total isolates (*n* = 167). ^a^ *Citrobacter freundii*, *Enterobacter cloacae*, *Escherichia coli*, and *Serratia marcescens*; ^b^ *Staphylococcus* spp. (*S. auricularis*, *S. hominis*, *S. saprophyticus*, *S. simulans*, *S. warneri*, *and S. xylosus*) *excluding S. pseudintermedius*, *S. capitis*, *S. epidermidis*, *Staphylococcus intermedius and S.* aureus. ^c^ *Gram-positive* and *Gram-negative bacteria* (this group was identified by Gram labeling since MALDI-TOF analysis yielded no identification) and *unknown* (this group was also not identified by MALDI-TOF analysis). These two groups likely represent multiple bacterial species on the target plate spot or no hit in the MALDI-TOF data base. ^d^ Presence of each isolate of 1% or less represented by *Acinetobacter johnsonii*, *Actinomyces* sp., *Bacillus pumilus*, *Bacillus* sp., *Exiguobacterium* sp., *Klebsiella oxytoca*, *Kocuria* sp., *Microbacterium* sp. *Micrococcus luteus, Moraxella canis*, *Pasteurella canis*, *Pseudomonas* sp., *Psychrobacter* sp., *Staphylococcus aureus*, *Streptococcus lutetiensis*, and *Streptococcus salivarius*.

*Organism*	(*n* = 167)
*Staphylococcus pseudintermedius*	22%
*Staphylococcus epidermidis*	12%
*Staphylococcus capitis*	11%
*Pseudomonas aeruginosa*	10%
*enteric Gram-negative rods* ^a^	7%
*coagulase negative staphylococci* ^b^	5%
*Streptococcus canis*	5%
*Corynebacterium* sp.	2%
*Enterococcus faecalis*	2%
*Streptococcus* sp.	2%
*Rothia* sp.	2%
*Enterococcus faecium*	1%
*Staphylococcus aureus*	1%
*Staphylococcus intermedius*	1%
*Other* ^c^	5%
*Other* ^d^	10%

**Table 2:** Resistance profile of isolates from corneal stromal ulcer samples from 2018–2021. Percentage (%) of resistant bacterial species from patient samples presented as combined resistance (TOTAL; intrinsic and acquired resistance together), intrinsic resistance only (INTRINSIC), or acquired resistance only (ACQUIRED). The number of isolates is 167 for each antibiotic in the TOTAL column exceptfor oxacillin, which numbered 29. * = although amoxicillin/clavulanic acid is not used topically, data for this antibiotic are presented here since it is often used postoperatively as a systemic antibiotic.

Antibiotic	Total	Intrinsic	Acquired
polymyxin B	60%	1%	59%
oxacillin	59%	0%	59%
cefoxitin	46%	7%	39%
cefazolin	46%	11%	35%
amoxicillin/clavulanic acid *	44%	10%	34%
tetracycline	38%	5%	33%
tobramycin	32%	5%	27%
neomycin	29%	10%	19%
bacitracin	26%	0%	26%
ofloxacin	23%	0%	23%
gentamicin	21%	4%	17%
amikacin	16%	6%	10%

**Table 3:** Bacterial species distribution from corneal stromal ulcer samples relative to the patient’s previous antibiotic treatments. Distribution of isolates in canine patients. Data are presented as a percentage of total isolates (*n* = 61 and *n* = 106). ^a^ *Citrobacter freundii, Enterobacter cloacae, Escherichia coli*, and *Serratia marcescens*; ^b^ *Staphylococcus* spp. (*S. auricularis*, *S. hominis*, *S. saprophyticus*, *S. simulans*, *S. warneri*, and *S. xylosus*) excluding *S. pseudintermedius*, *S. capitis*, *S. epidermidis*, *Staphylococcus intermedius* and *S. aureus*. ^c^ Gram-positive and Gram-negative bacteria (this group was identified by Gram labeling since MALDI-TOF analysis yielded no identification) and unknown (this group was also not identified by MALDI-TOF analysis). These two groups likely represent multiple bacterial species on the target plate spot or no hit in the MALDI-TOF data base. ^d^ Presence of each isolate of 1% or less represented by *Acinetobacter johnsonii*, *Actinomyces* sp., *Bacillus pumilus*, *Bacillus* sp., *Exiguobacterium* sp., *Klebsiella oxytoca*, *Kocuria* sp., *Microbacterium* sp. *Micrococcus luteus*, *Moraxella canis*, *Pasteurella canis*, *Pseudomonas* sp., *Psychrobacter* sp., *Streptococcus lutetiensis*, and *Streptococcus salivarius*.

*Organism*	No Previous Antibiotic Treatment (*n* = 61)	Previous Antibiotic Treatment (*n* = 106)
*Staphylococcus pseudintermedius*	34%	15%
*Staphylococcus epidermidis*	2%	18%
*Staphylococcus capitis*	8%	13%
*Pseudomonas aeruginosa*	15%	8%
*enteric Gram-negative rods* ^a^	7%	7%
*coagulase negative staphylococci* ^b^	7%	5%
*Streptococcus canis*	5%	6%
*Corynebacterium* sp.	0%	3%
*Enterococcus faecalis*	2%	2%
*Streptococcus* sp.	3%	1%
*Rothia* sp.	2%	3%
*Enterococcus faecium*	0%	2%
*Staphylococcus aureus*	0%	2%
*Staphylococcus intermedius*	3%	0%
*Other* ^c^	3%	7%
*Other* ^d^	10%	10%

**Table 4:** Distribution of the most common bacterial species from canine corneal ulcers across various geographical locations.

Location	Author, Year	*Staphylococcus* spp.	*Pseudomonas aeruginosa*	*Streptococcus* spp.
Australia	Hindley et al., 2015 [7]	18%	31%	31%
Taiwan	Lin et al., 2007 [25]	49%	8%	7%
Thailand	Ekapopphan et al., 2018 [11]	46%	21%	8%
UK	Tsvetanova et al., 2020 [10]	14%	40%	28%
Switzerland	Suter et al., 2018 [8]	41%	11%	26%
Brazil	Prado et al., 2005 [24]	57%	5%	11%
Brazil	Varges et al., 2009 [26]	59%	-	-
Southeast US	Tolar et al., 2006 [6]	33%	21%	17%
Southeast US	McKeever, 2021 [5]	34%	18%	28%
Midwest US	Jinks et al., 2020 [12]	36%	10%	34%
Midwest US	Hewitt et al., 2020 [15]	32%	12%	19%
Midwest US	this study, 2022	50%	10%	7%

**Table 5:** Comparison of susceptibility profiles of isolates in the midwestern US between Hewitt et al. and this study.

**Topical Antibiotic Susceptibility**		
**Antibiotic**	**Hewitt et al.**	**This Study**
amikacin	77%	84%
amikacin and cefazolin	79%	93%
bacitracin	7%	74%
cefazolin	8%	54%
gentamicin	74%	79%
gentamicin and cefazolin	76%	90%
gentamicin and ofloxacin	87%	88%
neomycin	76%	71%
neopoly	76%	79%
neopolybac	77%	96%
ofloxacin	53%	77%
ofloxacin and cefazolin	55%	87%
polymyxin B	0%	40%
tobramycin	57%	68%
**Systemic Antibiotic Susceptibility**		
amoxicillin/clavulanic acid	78%	56%
cephalexin	23%	35%
clindamycin	61%	41%
doxycycline	56%	66%
enrofloxacin	64%	79%
marbofloxacin	75%	85%
penicillin	35%	26%
trimethoprim/sulfamethoxazole	53%	59%

## Data Availability

The data presented in this study are available on request from the corresponding author. The data are not publicly available due to privacy concerns (client identification information).
